# Hypoxia Stimulates the EMT of Gastric Cancer Cells through Autocrine TGFβ Signaling

**DOI:** 10.1371/journal.pone.0062310

**Published:** 2013-05-17

**Authors:** Junko Matsuoka, Masakazu Yashiro, Yosuke Doi, Yuhiko Fuyuhiro, Yukihiro Kato, Osamu Shinto, Satoru Noda, Shinichiro Kashiwagi, Naoki Aomatsu, Toshiki Hirakawa, Tsuyoshi Hasegawa, Kiyoshi Shimizu, Toshiyuki Shimizu, Atsushi Miwa, Nobuya Yamada, Tetsuji Sawada, Kosei Hirakawa

**Affiliations:** 1 Department of Surgical Oncology, Osaka City University Graduate School of Medicine, Abeno-ku, Osaka, Japan; 2 Oncology Institute of Geriatrics and Medical Science, Osaka City University Graduate School of Medicine, Abeno-ku, Osaka, Japan; 3 Pharmacological Research Laboratories, Kyowa Hakko Kirin Co., Ltd., Chiyodaku, Tokyo, Japan; 4 Research Planning Department, Kyowa Hakko Kirin Co., Ltd., Chiyodaku, Tokyo, Japan; 5 Biologics Research Laboratories, Kyowa Hakko Kirin Co., Ltd., Chiyodaku, Tokyo, Japan; University of São Paulo, Brazil

## Abstract

Epithelial mesenchymal transition (EMT) is considered to be correlated with malignancy of cancer cells and responsible for cancer invasion and metastasis. We previously reported that distant metastasis was associated with hypoxia in gastric cancer. We therefore investigated the effect of hypoxic condition on EMT of gastric cancer cells. Gastric cancer cells were cultured in normoxia (21% O_2_) or hypoxia (1% O_2_) for 24 h. EMT was evaluated as the percentage of spindle-shaped cells in total cells. Effect of transforming growth factor β1 (TGFβ1) or tyrosine kinase inhibitors on the EMT was evaluated. The expression level of *TGFβ1* and *TGFβR* was evaluated by real time RT-PCR. The TGFβ1 production from cancer cells was measured by ELISA. Hypoxia stimulated EMT of OCUM-2MD3 and OCUM-12 cells, but not that of OCUM-2M cells. The expression level of *TGFβ1* mRNA under hypoxia was significantly higher than that under normoxia in all of three cell lines. The expression level of *TGFβR* mRNA was significantly increased by hypoxia in OCUM-2MD3 cells, but not in OCUM-2M cells. TGFβR inhibitor, SB431542 or Ki26894, significantly suppressed EMT of OCUM-2MD3 and OCUM-12. TGFβ1 production from OCUM-2MD3 and OCUM-12 cells was significantly increased under hypoxia in comparison with that under normoxia. These findings might suggest that hypoxia stimulates the EMT of gastric cancer cells via autocrine TGFβ/TGFβR signaling.

## Introduction

Epithelial mesenchymal transition (EMT) is characterized by changes in cell morphology during which epithelial cells acquire mesenchymal properties while losing cell-cell interactions and apicobasal polarity [1], [2]. In epithelial cancers, EMT is recognized as one of the mechanisms responsible for initiating the invasive and metastatic behaviors [3]–[5].

A hypoxic environment exists in some regions of solid cancers that show rapid growth because angiogenesis in carcinomas is heterogeneous [6]. Hypoxia is considered to be associated with aggressive tumor phenotypes of gastric carcinomas [7], [8], including metastatic ability of cancer cells [6], [9]. Clinical and experimental data also provide evidence of an association between the hypoxic environment and poor prognosis [10], [11]. It is thus important for the future development of cancer treatments to clarify the mechanism of metastasis induced by hypoxia.

It has been reported that various soluble factors, including transforming growth factor-β1 (TGFβ1) [12], [13], epidermal growth factor (EGF) [14], insulin-like growth factor-1 (IGF1) [15], fibroblast growth factor (FGF) [16], hepatocyte growth factor (HGF) [17] were correlated with EMT. TGFβ signals have an important role in various aspects of the metastatic spread of cancer cells, such as migration, invasion, and EMT [12], [13]. The TGFβ ligands bind to TGFβ receptor type II (TGFβRII), which then forms a complex with either TGFβR type I or II. TGFβR type I (TGFβRI) transmits signals within the cell via second-messenger Smad proteins [13], [18]. Downstream signals are propagated through TGFβRI, which phosphorylates receptor-regulated Smad proteins [19], [20].

Several studies have reported that a hypoxic condition might induce EMT of cancer cells [21]–[24], but the molecular mechanism responsible for EMT under a hypoxic condition remains unclear. We therefore investigated the effect of hypoxia on the morphologic characteristics of gastric cancer cells to clarify the mechanisms responsible for hypoxia-induced EMT.

## Materials and Methods

### Cell Culture and Cell lines

Seven gastric cancer cell lines were used. OCUM-2MD3 [25], OCUM-12 [26], OCUM-2M [27], KATO-III [28], and MKN-45 [29] were derived from diffuse-type gastric carcinoma. MKN-74 [29] and MKN-7 [29] were derived from intestinal-type. OCUM-2M, OCUM-2MD3 and OCUM-12 were establised in our laboratory, as previously reported [25]–[27]. Briefly, OCUM-12was derived from ascites associated with diffuse-type gastric carcinoma, and OCUM-2MD3 cells, a daughter cell line with high potential of peritoneal metastasis, were established from OCUM-2M cells using orthotopic tumor model, a parental cell line ascites associated with diffuse-type gastric carcinoma. The other cell lines were obtained from the JCRB cell bank (Osaka, Japan) or the American Type Culture Collection (Rockville, MD). Cells were cultured at 37°C in 21% O_2_ (normoxia) or 1% O_2_ (hypoxia). Hypoxic conditions were maintained using a modular incubator chamber (Hirasawa, Tokyo, Japan) with 5% CO_2_ and 1% O_2_ balanced with N_2_ gas. The culture medium consisted of Dulbecco's modified Eagle medium (DMEM; Nikken Bio., Osaka, Japan) with 10% fetal bovine serum (Nichirei Bio.), 100 IU/ml penicillin (ICN Biomedicals, Costa Mesa, CA), 100 μg/ml streptomycin (ICN Biomedicals), and 0.5 mM sodium pyruvate (Cambrex, Walkersville, MD).

### Morphological changes

Cancer cells were cultured under normoxic or hypoxic conditions for 24 h, and cell morphology was observed microscopically. EMT was determined when the polygonal or spindle shape was found in cancer cells by phase-contrast microscope. The frequency of EMT was evaluated by the rate of polygonal or spindle-shaped cells in all cancer cells; EMT rate  =  the number of polygonal or spindle shape cells/total number of cells ×100 (%).

### Effects of various soluble factors on morphological changes

We examined the effects of various soluble factors, including EGF (Pepro Tec, Rocky Hill, NJ), IGF1 (Pepro Tec), vascular endothelial cell growth factor (VEGF; R&D systems, Minneapolis, MN), basic-FGF (Sigma, Saint Louis, MO), platelet-derived growth factor BB homodimer (PDGF-BB; Pepro Tech), HGF (Pepro Tech), TGFβ1 (King Brewing, Kakogawa, Japan), on the morphology of cancer cells. Cells were cultured in DMEM containing one of above factors at the required concentration at 37°C in normoxia. Cell morphology was examined after 24 h. Experiments were performed for each factor in duplicate.

### Effects of neutralizing antibodies on morphological changes

We used neutralizing antibodies, such as anti-HGF antibody (Genzyme techne, MPLS, MN, USA), anti-TGFβ antibody (R&D systems), anti-PDGF-BB (Genzyme), anti-FGF7 (R&D systems), anti-VEGFR3 (R&D systems). Cells were cultured in DMEM containing one of above antibodies at 100 μg/ml at 37°C in hypoxia. Cell morphology was examined after 24 h.

### Effect of various phospholylation inhibitors on morphological changes

After cells reached semi-confluence, cells were added to TGFβ or each inhibitor and incubated under hypoxic condition or normoxia for 24 h. Then, morphologic findings were examined in comparison with the control. Five small-synthetic phospholylation inhibitors, Ki26894 (TGFβRI inhibitor, 10 μM; Kirin Pharma, Mishima, Japan), SB431542 (TGFβRI inhibitor, 10 μM; Sigma, Saint Louis, MO), Sunitinib (SU11248, 20 nM; a tyrosine kinase inhibitor against VEGFR, PDGFR, c-KIT, FGFR2 and FLT3, LKT Laboratories, St. Pail, MN), PD173074 (FGF/VEGF receptor tyrosine kinase inhibitor, 5 nM; Santa Cruz, CA), Lapatinib (GW572016, 10 nM; a tyrosine kinase inhibitor against EGFR and ErbB2, Toronto Research Chemicals), and PHA665752 (c-MET inhibitor, 2 μM; Pfizer, San Diego, CA, USA) were used.

### Quantitative real-time reverse transcriptase-polymerase chain reaction (RT-PCR)

Quantitative RT-PCR was performed to examine *TGFβ1*, *TGFβRI, TGFβRII,* and *Vimentin* mRNA expressions. Cancer cells were incubated under normoxic conditions and under hypoxic conditions for 24 h. After incubation, the total cellular RNA was extracted using Trizol reagent (Invitrogen, Carlsbad, CA) according to the manufacturer's instructions. After removal of genomic DNA by DNase, cDNA was prepared from 2 μg RNA with Maloney mouse leukemia virus reverse transcriptase (Invitrogen) using random primers (Invitrogen). To determine fold changes in each gene, real-time RT-PCR was performed on the ABI Prism 7000 (Applied Biosystems, Foster City, CA), using commercially available gene expression assays for *TGFβ1* (Hs00998130,), *TGFβRI* (Hs00610319), *TGFβRII* (Hs00559661), *vimentin* (Hs00958116), *Twist* (Hs01675818), *Zeb1* (Hs 00232783), *Snail1* (Hs00195591), *VEGFA* (Hs00900055). PCR was performed at 95°C for 15 s and 60°C for 60 s for 40 cycles. Glyceraldehyde-3-phosphate dehydrogenase (GAPDH) was used as an internal standard to normalize mRNA levels for differences in sample concentration and loading. Fold changes in the expression of each target mRNA relative to GAPDH was calculated based on the threshold cycle (Ct) as 2^−Δ (ΔCt)^, where ΔCt  =  Ct target- Ct GAPDH and Δ (ΔCt)  = ΔCt_hypoxia_−ΔCt_normoxia_. Quantitative PCR reactions were performed in triplicate.

### Western blot analysis

For examining the effect of hypoxia on Smad2 phosphorylation, cancer cells were incubated under normoxic or hypoxic conditions for 24 h, respectively. The cells were lysed in a lysis buffer, and aliquots containing 50 μg of total protein were subjected to sodium dodecyl sulfate-polyacrylamide gel electrophoresis; the protein bands were transferred to a polyvinylidene difluoride membrane (Millipore, Billerica, MA). The membrane was incubated in TBS-T (10 mM TBS and 0.05% Tween 20) supplemented with 5% non-fat milk or 5% bovine albumin (Sigma) at room temperature for 1 h. Next, the membrane was placed in a TBS-T solution containing the primary antibody p-Smad2 (Ser^465/467^; 1:1000; Cell Signaling Technology) or Smad2/3 (1:1000; Cell Signaling Technology), Vimentin (1:1000; Cell Signaling Technology), anti-βactin (1:1000; Sigma) and allowed to react at 4°C overnight. Then, each antibody was washed three times with TBS-T for 10 min, and a peroxidase-labeled secondary antibody (GE healthcare, Buckinghamsire, UK) reactive with the primary antibody was added. The bands were detected using an enhanced chemiluminescence system (Wako, Osaka, Japan).

### Enzyme-linked immunosorbent assay (ELISA)

The TGFβ1 production from cancer cells was measured by a quantitative sandwich enzyme immunoassay technique using a Quantikine human TGFβ1 ELISA kit, according to the manufacturer`s instruction (R&D systems). Cancer cells were incubated under hypoxia or normoxia for 24 h, then the medium was replaced to 3 ml serum free DMEM. Cells were incubated under hypoxia or normoxia, for additional 24 h. Conditional medium (CM) was collected from each dish and centrifuged at 1000 *g* for 5 min. TGFβ1 level of serum free CM was measured using ELISA kit. ELISA detected both the active and latent TGFβ.

### Statistical analysis

Comparisons among data sets were made with Student's *t*-test or the Kruskal-Wallis one-way ANOVA by ranks followed by Dunn's multiple comparison test. Differences were considered to be statistically significant when the P value was <0.05.

## Results

### Effects of hypoxia on the cancer cell morphology

**Figure 1:**
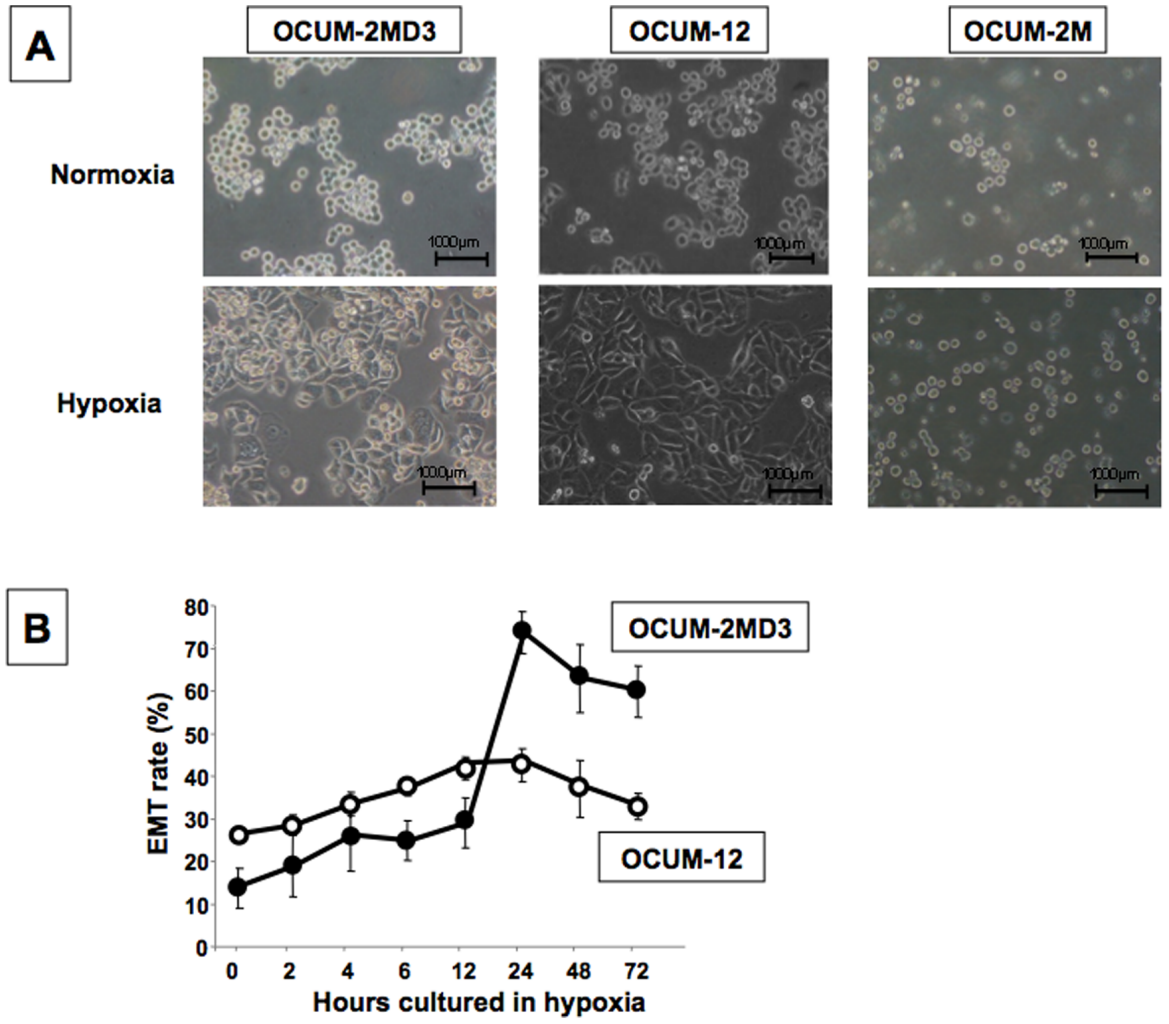
Morphologic changes of gastric cells under a hypoxic condition. (A) An increase of polygonal or spindle-shape cells was recognized in OCUM-2MD3 and OCUM-12 cells under hypoxia, but not in OCUM-2M cells. (B) The morphology of OCUM-2MD3 and OCUM-12 cells began to change after 4 h in hypoxic culture. EMT of OCUM-2MD3 and OCUM-12 cells was most evident at 24 h of culture under hypoxia. EMT was determined when the polygonal or spindle shape was found in cancer cells by phase-contrast microscope.

A hypoxic condition significantly increased the number of polygonal or spindle-shaped cells undergoing EMT among OCUM-2MD3 or OCUM-12 cells, but not among OCUM-2M, MKN-7, MKN-45, MKN-74, or KATO-III cells (Fig. 1A and Fig. S1). The cell morphology of OCUM-2MD3 and OCUM-12 began changing after 4 h in the hypoxic culture. After 12 h of culture, an increased rate of EMT was seen in both cell lines. The EMT rate of OCUM-2MD3 and OCUM-12 cells was highest at 24 h hypoxic culture (Fig. 1B). Since the morphologic changes were evident at 24 h of culture under hypoxia, the molecular mechanisms of EMT were analyzed at 24 h of culture under hypoxic conditions using OCUM-2MD3, OCUM-12, and OCUM-2M cells.

### Effects of various growth factors on cancer cell morphology

**Figure 2:**
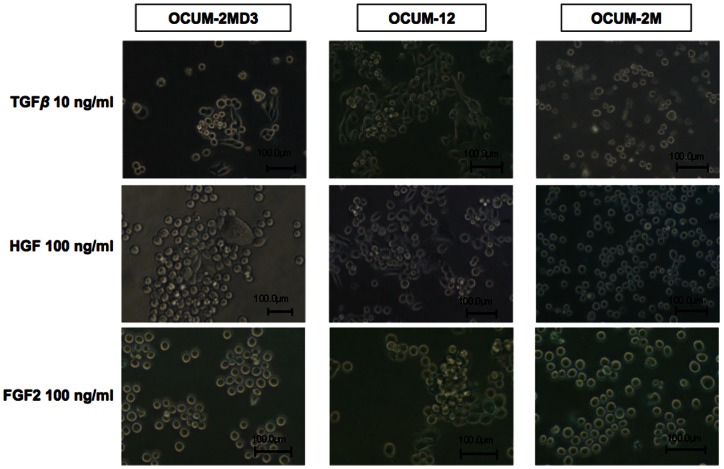
Effects of various growth factors on cancer cell morphology. Morphologic change was found in the presence of TGFβ and HGF at 24 h of culture in OCUM-2MD3 and OCUM-12 cells, but not FGF2.

Soluble factors were used at concentrations of 10 or 100 ng/ml. OCUM-2MD3 and OCUM-12 cells became spindle-shaped following the addition of TGFβ1 or HGF, but not following the addition of EGF, FGF2, FGF7, IGF1, VEGF or PDGF-BB after 24 h under normoxia (Table 1). In contrast, OCUM-2M cells showed no morphologic change following the addition of any factors (Fig. 2).

**Table 1:** Effects of various growth factors on cancer cell morphology.

Growth Factor	OCUM-2MD3		OCUM-12			OCUM-2M
	concentration		concentration			concentration
	10 ng/ml	100 ng/ml	10 ng/ml	100 ng/ml	10 ng/ml	100 ng/ml
TGFβ1	+	+	+	+	−	−
HGF	−	+	−	+	−	−
FGF2	−	−	−	−	−	−
FGF7	−	−	−	−	−	−
EGF	−	−	−	−	−	−
IGF1	−	−	−	−	−	−
VEGF	−	−	−	−	−	−
PDGF-BB	−	−	−	−	−	−

+; Positive morphologic changes, −; No changes.

The culture medium was consisted of DMEM with 10% FBS.

### Effects of neutralizing antibodies on cancer cell morphology under hypoxia

EMT induced by hypoxia was partly inhibited by anti-TGF-β1 antibody at 100 μg/ml, but not by anti-HGF antibody (Table 2).

**Table 2:** Effects of neutralizing antibodies on cancer cell morphology under hypoxia.

Neutralizing antibody	OCUM-2MD3	OCUM-12
anti-TGFβ antibody (100 μg/ml)	+	+
anti-HGF antibody (100 μg/ml)	−	−

Effects of neutralizing antibodies were determined after 24 h of culture.

+; Partly effective, −; Not effective.

The culture medium was consisted of DMEM with 10% FBS.

### Effects of hypoxia on *TGFβ1*, *TGFβRI*, and *TGFβRII* mRNA expression of gastric cancer cells

**Figure 3:**
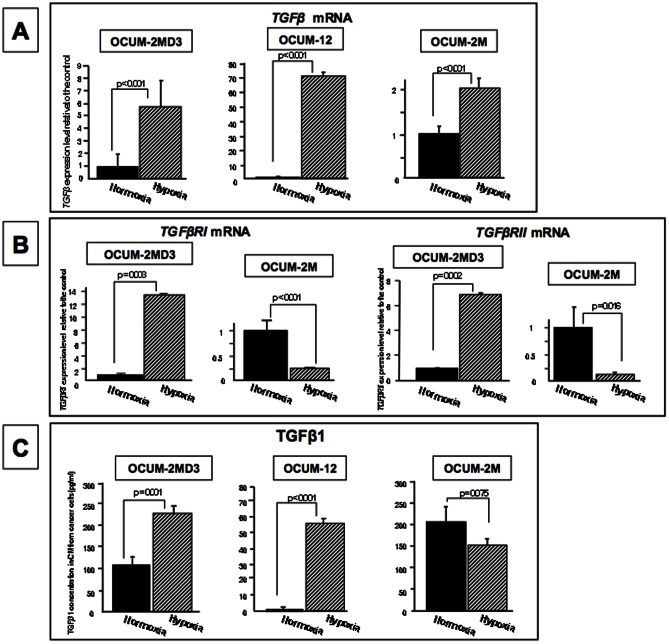
Effects of hypoxia on the expressions of *TGFβ* and *TGFβR* and the production of TGFβ1 in cancer cells. (A) *TGFβ* mRNA expression was significantly (p<0.001) increased under a hypoxic condition in OCUM-2M, OCUM2MD3 and OCUM-12 cells. (B) The expression levels of *TGFβRI* and *TGFβRII* were significantly (p<0.001) increased under a hypoxic condition in OCUM-2MD3, but not in OCUM-2M cells. (C) TGFβ1 production in OCUM-2MD3 and OCUM-12 cells was significantly (p = 0.001 and p<0.001) higher under hypoxia (232 pg/ml, 80 pg/m, respectively) than under normoxia (109 pg/ml, 34 pg/ml, respectively). TGFβ1 production in OCUM-2M cells was not different between normoxia and hypoxia.

The expression level of *TGFβ* was significantly (p<0.001) increased under a hypoxic condition in all of OCUM-2MD3, OCUM-12, and OCUM-2M cells, in comparison with the level under normoxia (Fig. 3A). The expression levels of *TGFβRI* and *TGFβRII* were significantly increased under a hypoxic condition in OCUM-2MD3 cells. In contrast, the levels of *TGFβR1* and *TGFβR2* expression were significantly decreased under a hypoxic condition in OCUM-2M cells (Fig. 3B).

### Effect of hypoxia on TGFβ1 production released from gastric cancer cells

TGFβ1 production from OCUM-2MD3 and OCUM-12 cells was significantly (p<0.001) increased under hypoxia compared to normoxia, but not that from OCUM-2M cells (Fig. 3C).

### Effects of hypoxia on Smad2 phosphorylation of gastric cancer cell lines

**Figure 4:**
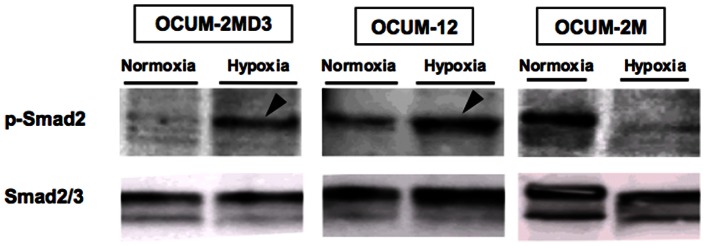
Smad2 phosphorylation of gastric cancer cells under a hypoxic condition. Hypoxic condition for 24 h increased Smad2 phosphorylation of OCUM-2MD3 and OCUM-12 cells (arrowheads), but not that of OCUM-2M cells. Smad2/3 was used as an international control for p-Smad2.

In OCUM-2MD3 and OCUM-12 cells, Smad2 phosphorylation was increased under a hypoxic condition, compared to that under a normoxic condition. In contrast, Smad2 phosphorylation was not increased under a hypoxic condition in OCUM-2M cells (Fig. 4).

### Effects of phosphorylation kinase inhibitors on EMT and vimentin expression in hypoxia

**Figure 5:**
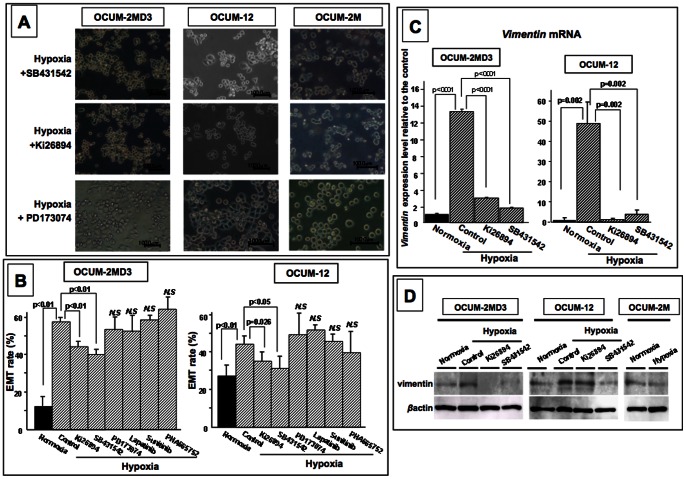
Effects of inhibitors on EMT under hypoxic condition. (A, B) Hypoxia-induced EMT was significantly (p<0.01) inhibited by TGFβR inhibitors (Ki26894 and SB431542). Other inhibitors (Lapatinib, Sunitinib, and PHA665752) had no effect on EMT under hypoxia. (C) *Vimentin* mRNA expression was significantly increased under a hypoxic condition in OCUM-2M and OCUM-12 cells, and decreased with TGFβR inhibitors (Ki26894 and SB431542). (D)Vimentin protein was increased under hypoxia in OCUM-12 and OCUM-2MD3, and decreased with TGFβR inhibitors (Ki26894 and SB431542).

Either of the TGFβR phosphorylation inhibitors, Ki26894 and SB431542, inhibited the morphological changes of OCUM-2MD3 and OCUM-12 cells under a hypoxic condition, but three other inhibitors, Lapatinib, Sunitinib, and PHA665752, did not (Figure 5A, 5B). On the other hand, none of the inhibitors had any effect on the morphology of OCUM-2MD3 and OCUM-12 cells under normoxia (data not shown). The expression level of *vimentin*, which was used as an EMT marker, was significantly increased by hypoxia, and was decreased by either of the TGFβR inhibitors Ki26894 and SB431542 (Figure 5C). Vimentin protein level was also increased under hypoxia, and was decreased by either of the TGFβR inhibitors Ki26894 and SB431542 (Figure 5D).

#### Effects of hypoxic condition on the factors associated with EMT

**Figure 6:**
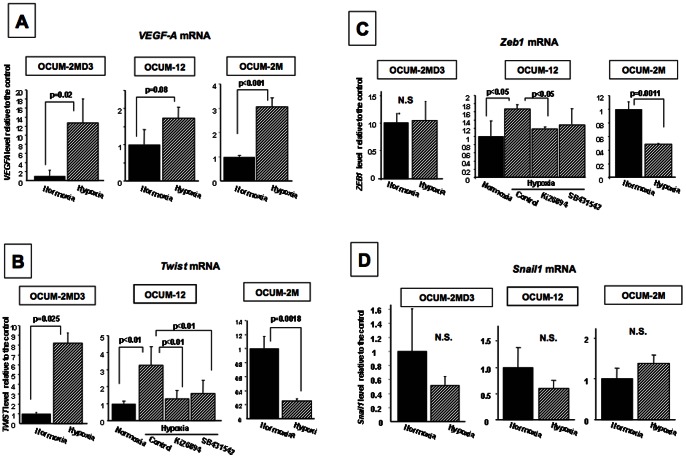
Effects of hypoxic condition on the transcription factors associated with mesenchymal transition. (A) A hypoxia-target molecule, *VEGF-A* mRNA level in OCUM-2M, OCUM-2MD3, and OCUM-12 was increased under hypoxia. (B, C) Hypoxic condition significantly increased the expression level of *Twist* and *Zeb1* in OCUM-2MD3 and OCUM-12 cells, but not that in OCUM-2M cells. (D) The expression level of Snail1 was not affected by hypoxic condition.

Hypoxic condition significantly increased *VEGF-A* mRNA level in OCUM-2M, OCUM-2MD3, and OCUM-12 cells. The expression level of *Twist* or *Zeb1* was significantly increased by hypoxia in OCUM-2MD3 and OCUM-12 cells, but was decreased in OCUM-2M cells. The increased expression of *Twist* or *Zeb1* was decreased by either of the TGFβR inhibitors Ki26894 and SB431542 in OCUM-12 cells. Hypoxic condition did not affected on the expression level of *Snail1* (Figure 6).

## Discussion

Recently, several studies have reported that a hypoxic microenvironment surrounding solid tumor cells might contribute to cancer progression [30]–[34]. However, the molecular mechanisms responsible for the cancer progression in hypoxia remain unclear. It has been reported that hypoxia is one of the triggers for EMT [35]. In the present study, a hypoxic condition induced EMT in the diffuse-type gastric cancer cell lines OCUM-2MD3 and OCUM-12, but not in intestinal-type cells. Diffuse-type gastric cancer is characterized by higher malignancy than intestinal-type gastric cancer [36]. The EMT potential of cancer cells under hypoxia might be one of the reasons for the high malignant potential of diffuse-type gastric cancer.

Many studies have reported that several molecules might affect EMT of cancer cells, including TGFβ [23], [37], EGF [38], and PDGF [39]. In the present study, TGFβ stimulated EMT of OCUM-2MD3 and OCUM-12 cells, but EGF and PDGF did not. In addition, EMT induction under a hypoxic condition was significantly decreased by anti-TGFβ neutralizing antibody or TGFβR phosphorylation inhibitors in both OCUM-2MD3 and OCUM-12 cells. Taken together, these results indicate that the production level of TGFβ in cancer cells was significantly increased under hypoxia, and the expression levels of TGFβR and Smad2 phosphorylation were increased by hypoxia. Vimentin is a characteristic component of mesenchymal cells and is not usually expressed in epithelial cells. Because the expression of *vimentin* in epithelial cells plays an essential role in EMT through the interaction with actin and other intermediate filaments [40], vimentin was used as an EMT marker in this study. The expression level of *vimentin* was significantly increased by hypoxia, and was decreased by the TGFβR phosphorylation inhibitors Ki26894 and SB431542. These findings suggested that EMT under a hypoxic condition is associated with the autocrine TGFβ/TGFβR signaling in diffuse-type gastric cancer. Several studies reported the correlations between EMT and TGFβ signaling in hypoxia. [2], [21], [23], [32], [37] In gastric cancer, it has been reported that EMT is correlated with malignancy of cancer cells and responsible for cancer invasion and metastasis. However the mechanisms responsible for EMT of gastric cancer cells remain unclear. This paper clarified that EMT of gastric cancer cells was induced via TGFβ signaling under hypoxia. TGFβR inhibitors might thus be promising agents for the treatment of gastric cancer metastasis.

Hypoxia stimulated EMT of OCUM-2MD3 cells, but not that of OCUM-2M cells. EMT has been implicated in promoting cancer invasion and metastasis. While OCUM-2MD3 is a “daughter” cell line of OCUM-2M [25], [34], OCUM-2MD3 cells have higher potential for metastasis to the peritoneum in nude mice than OCUM-2M cells [25], [34]. Cancer cells free to migrate in the peritoneal cavity are exposed to hypoxia due to the lack of a proximal feeding vessel [41]. The expression level of *TGFβ1* mRNA under hypoxia was significantly higher than that under normoxia in all three of the cell lines. In contrast, the expression level of *TGFβRI* and *TGFβRII* mRNA was significantly increased by hypoxia in OCUM-2MD3 cells, but not in OCUM-2M cells. The different responses of *TGFβR* expression between normoxia and hypoxia might be one of the key reasons for the hypoxia-induced EMT. We previously reported that gastric cancer cells under hypoxia showed EMT and high migratory and invasive activities in comparison with cancer cells in normoxia [34]. The up-regulation of EMT of hypoxic cancer cells might be one of the reasons for the higher potential for metastasis to the peritoneum in OCUM-2MD3 cells compared to OCUM-2M cells.

Hypoxic condition significantly increased the expression level of *Twist* and *Zeb1* in OCUM-2MD3 and OCUM-12 cells, but not that in OCUM-2M cells. The hypoxia-increasing activity of *Twist* and *Zeb1* expression was decreased with TGFβR inhibitors (Ki26894 and SB431542) in OCUM-12 cells. EMT in hypoxia might be partly regulated by transcription factors, *Twist* and *Zeb1.*

Although HGF affected the cancer cell morphology in OCUM-2MD3 and OCUM-12 cells, hypoxia-induced EMT was not inhibited by anti-HGF neutralizing antibody or c-Met inhibitor in either OCUM-2MD3 or OCUM-12 cells. HGF production was not detected in OCUM-2MD3 or OCUM-12 cells under either hypoxia and normoxia (data not shown). Most of HGF is derived from stromal cells in gastric cancer [42]. While HGF might not play an important role for hypoxia-induced EMT, HGF might stimulate EMT of cancer cells via the cancer-stromal interaction.

The hypoxia-induced EMT of OCUM-2MD3 cells was partly inhibited by TGFβR inhibitors, but various other inhibitors, including c-Met inhibitor, PDGF inhibitor, EGFR inhibitor, and VEGFR inhibitor, had no effect on the hypoxia-induced EMT in this study. These findings suggest that other factor(s) might be associated with hypoxia-induced EMT in some cancer cells. In future studies, it will be necessary to examine other new factors which can alter cancer morphology.

In conclusion, the autocrine TGFβ/TGFβR signaling under hypoxia might be one of the factors associated with the aggressive phenotype of EMT in gastric carcinoma cells. TGFβ/TGFβR signaling inhibitors appear to be therapeutically promising in gastric cancer.

## Supporting Information

Figure S1 **Morphologic changes of gastric cells under a hypoxic condition.** In MKN-7, MKN-45, MKN-74, and KATO-III, morphologic changes were not found under hypoxia.
(TIF)
