# Supplementary figures and images for: Hypoxia Stimulates the EMT of Gastric Cancer Cells through Autocrine TGFβ Signaling
Source: PLoS One. 2013 May 17;8(5):e62310. doi: 10.1371/journal.pone.0062310 (PMC3656884; doi:10.1371/journal.pone.0062310)

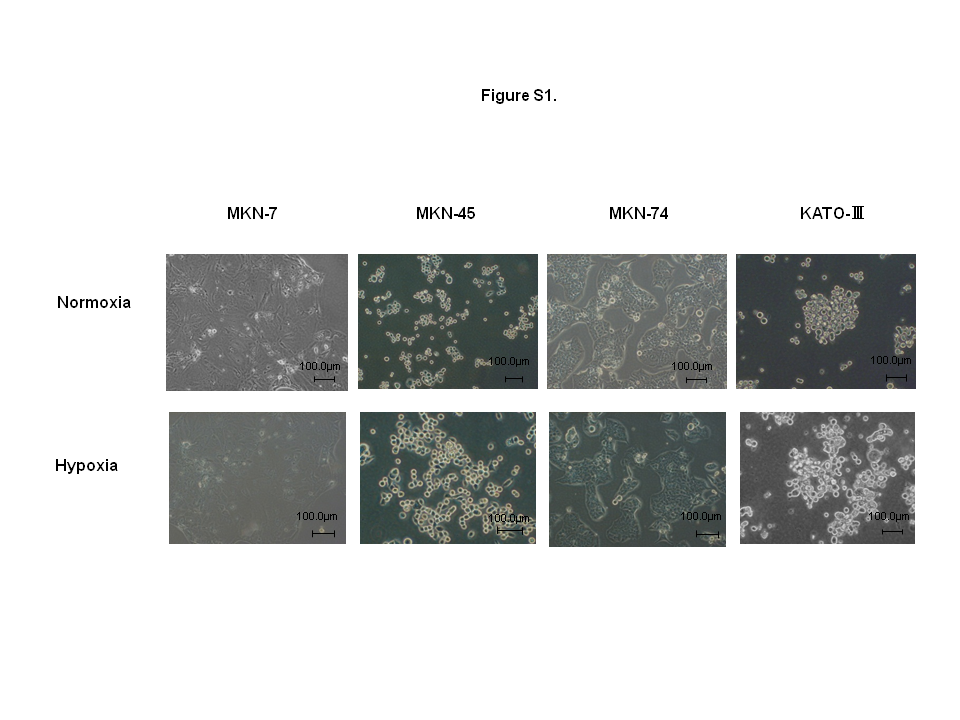

Supplement: Figure S1 — Morphologic changes of gastric cells under a hypoxic condition. In MKN-7, MKN-45, MKN-74, and KATO-III, morphologic changes were not found under hypoxia. (TIF) [file pone.0062310.s001.tif]
